# Anatomical landmarks for safer carpal tunnel decompression: an experimental cadaveric study

**DOI:** 10.1186/1754-9493-8-8

**Published:** 2014-02-17

**Authors:** Lasitha B Samarakoon, Malith H Guruge, Madusha Jayasekara, Ajith P Malalasekera, Dimonge J Anthony, Rohan W Jayasekara

**Affiliations:** 1 General surgical unit, National Hospital Sri Lanka, Colombo, Sri Lanka; 2 Department of Anatomy, Faculty of medicine, University of Colombo, Colombo, Sri Lanka

**Keywords:** Carpal tunnel decompression, Transverse carpal ligament, Recurrent branch, Palmar cutaneous branch, Superficial palmar arch, Avascular area

## Abstract

**Background:**

Carpal tunnel syndrome is a common presentation to surgical outpatient clinics. Treatment of carpal tunnel syndrome involves surgical division of the flexor retinaculum. Palmar and recurrent branches of the median nerve as well as the superficial palmar arch are at risk of damage.

**Methodology:**

Thirteen cadavers of Sri Lankan nationality were selected. Cadavers with deformed or damaged hands were excluded. All selected cadavers were preserved with the conventional arterial method using formalin as the main preservative. Both hands of the cadavers were placed in the anatomical position and dissected carefully. We took pre- determined measurements using a vernier caliper. We hypothesized that the structures at risk during carpal tunnel decompression such as recurrent branch of the median nerve and superficial palmar arch can be protected if simple anatomical landmarks are identified. We also hypothesized that an avascular area exists in the flexor retinaculum, identification of which facilitates safe dissection with minimal intra operative bleeding. Therefore we attempted to characterize the anatomical extent of such an avascular area as well as anatomical landmarks for a safer carpal tunnel decompression.

Ethical clearance was obtained for the study.

**Results:**

In a majority of specimens the recurrent branch was a single trunk (n =20, 76.9%). Similarly 84.6% (n = 22) were extra ligamentous in location. Mean distance from the distal border of the TCL to the recurrent branch was 7.75 mm. Mean distance from the distal border of TCL to the superficial palmar arch was 11.48 mm. Mean length of the flexor retinaculum, as measured along the incision, was 27.00 mm. Mean proximal and distal width of the avascular area on TCL was 11.10 mm and 7.09 mm respectively.

**Conclusion:**

We recommend incision along the radial border of the extended ring finger for carpal tunnel decompression. Extending the incision more than 8.16 mm proximally and 7.75 mm distally from the corresponding borders of the TCL should be avoided. Incision should be kept to a mean length of 27.0 mm, which corresponds to the length of TCL along the above axis. We also propose an avascular area along the TCL, identification of which minimizes blood loss.

## Introduction

Carpal tunnel syndrome (CTS) is a compression syndrome affecting the median nerve as it traverses under the flexor retinaculum on its way to the hand from the forearm.

Carpal tunnel syndrome causes pain, numbness, and tingling in the affected hand and is an important cause of work disability. In one study it was noted that symptoms of pain, numbness, and tingling in the hands are fairly common in the general population affecting 14.4% of the general population, with 1 in 5 symptomatic subjects expected to have carpal tunnel syndrome based on clinical examination and electrophysiologic testing [1]. Certain occupations have an increased risk of carpal tunnel syndrome than the general population. According to one study the greatest incidence was in caring and leisure occupations (197 per 100 000 population per year), while the lowest incidence was in the associate professional group (37 per 100 000). Professional occupations had a high incidence of carpal tunnel syndrome, along with skilled trades and elementary occupations. Women had a higher incidence of carpal tunnel syndrome than men in managerial, professional, skilled trades, and elementary occupations [2]. Although it may not be immediately obvious, carpal tunnel syndrome may have an immense adverse impact on the quality of life of affected patients [3].

Compression of the median nerve can be due to a multitude of causes. Among the commoner ones are trauma and fractures of carpal bones, arthritic conditions affecting the wrist and pregnancy. Rare cases of lipofibrohamartoma in the carpal tunnel with associated median nerve palsy have been described [4]. A case of bilateral Palmaris profundus tendons within the carpal tunnel, impinging on the median nerve leading to carpal tunnel syndrome has been reported [5]. Acute carpal tunnel syndrome associated with Haemophillia [6], as well as persistent median artery have also been noted in the literature [7]. A bifid median nerve is another rare anatomical entity leading to carpal tunnel syndrome [8].

Treatment of carpal tunnel syndrome can be medical or surgical. Steroid injection has been shown to be an appropriate treatment in carefully selected patients, although females and diabetics have been shown to have a higher risk of relapse [9]. Surgical treatment involves division of the flexor retinaculum, releasing the nerve. This can be achieved via traditional open decompression or recently developed mini incision techniques [10,11].

Even though considered to be a minor operation, carpal tunnel decompression can be associated with significant morbidity. In one study complications were noted to be around 12%. Complications were categorized into seven sub groups in the above study: (1) inadequate section of the transverse carpal ligament (2) damage to the palmar cutaneous branch of the median nerve, (3) reflex sympathetic dystrophy, (4) unsightly hypertrophic scar, (5) damage to the superficial palmar arch (6) bowstringing of the flexor tendons after excision of the transverse carpal ligament, and (7) adherence of the flexor tendons following excision of the mesotenon [12] in another study, 19% patients complained of Tender scars and 4% were affected by pillar pain. Grip strength was reduced in over 50% of the operated hands. Anaesthesia in the scar area were affecting 7% while 18% reported that there was incomplete relief of primary symptoms [13].

Complications can occur during open and endoscopic decompression. A case of iatrogenic damage to median nerve as well superficial palmar arch leading to pseudo aneurysm formation have been reported following endoscopic decompression [14].

Palmar cutaneous branches and of the median nerve have also been shown to be major hazards during carpal tunnel decompression [15]. Although commonly extra ligamentous, intra and pre ligamentous thenar branches of median nerve have been shown to be rare but real hazards during carpal tunnel decompression [16]. As noted above with regards to endoscopic approach, transection of the superficial palmar arch is a possible complication of open, limited incision carpal tunnel decompression [17].

## Methodology

A cadaveric study was carried out in order to determine anatomical land marks aiding the surgeon to perform a safer carpal tunnel decompression. Ethical clearance was obtained from ethics clearance committee (ERC) of faculty of medicine, university of Colombo. We hypothesized that the structures at risk during carpal tunnel decompression such as recurrent branch of the median nerve and superficial palmar arch can be protected if simple anatomical landmarks are identified. We also hypothesized that an avascular area exists in the flexor retinaculum, identification of which facilitates safe dissection with minimal intra operative bleeding. Therefore we attempted to characterize the anatomical extent of such an avascular area as well as anatomical landmarks for a safer carpal tunnel decompression.

Thirteen cadavers (8 male and 5 female) of Sri Lankan nationality were selected randomly regardless of the age. Cadavers with deformed or damaged hands were excluded from the study. All selected cadavers were preserved with the conventional arterial injection method using formalin as the main preservative. Both hands (26 all together) of the selected cadavers were placed in the anatomical position and dissected carefully to identify the anatomical structures. Pre- determined measurements were taken using a Vernier caliper. [Manufacturer- Mitutoyo (Kanagawa- Japan) [Model No- 505-633-50].

**Figure 1:**
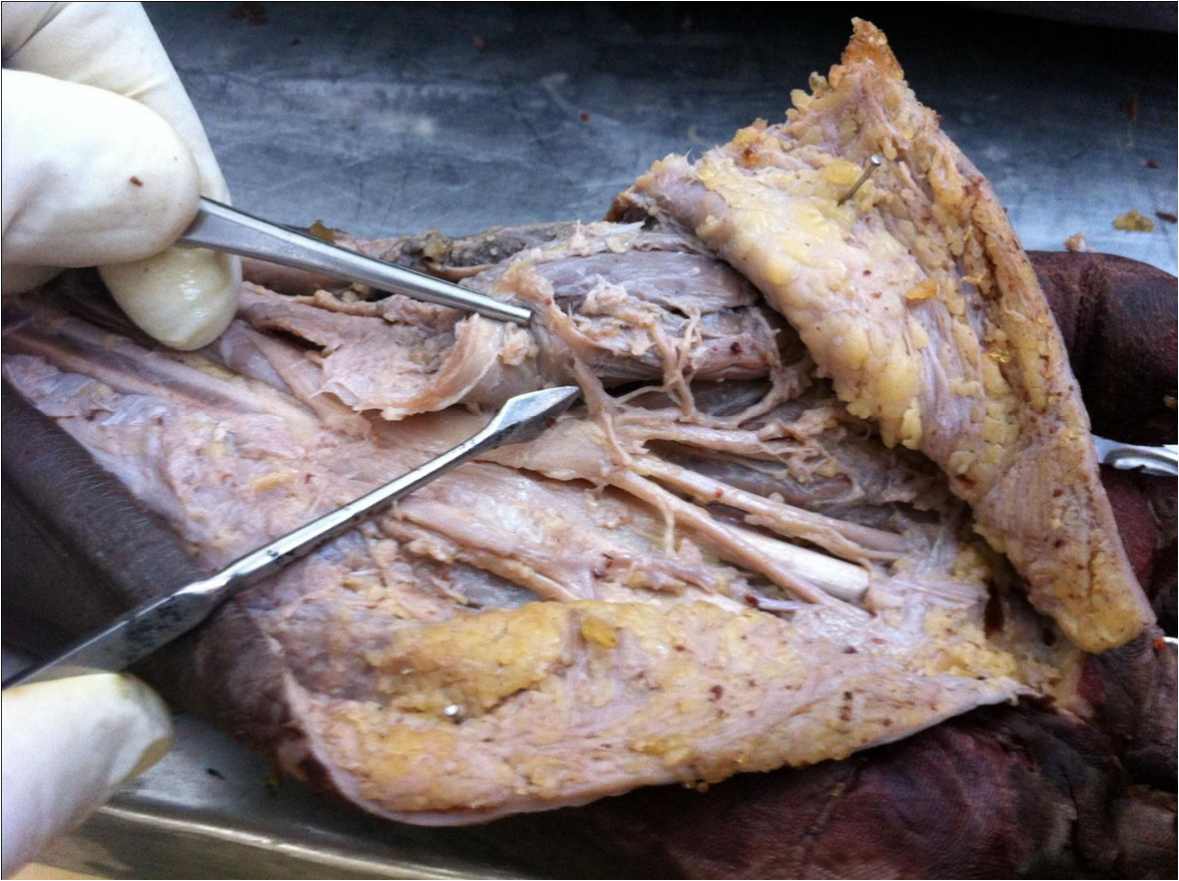
Dissected specimen showing an intra ligamentous recurrent branch of the median nerve (pointer).

**Figure 2:**
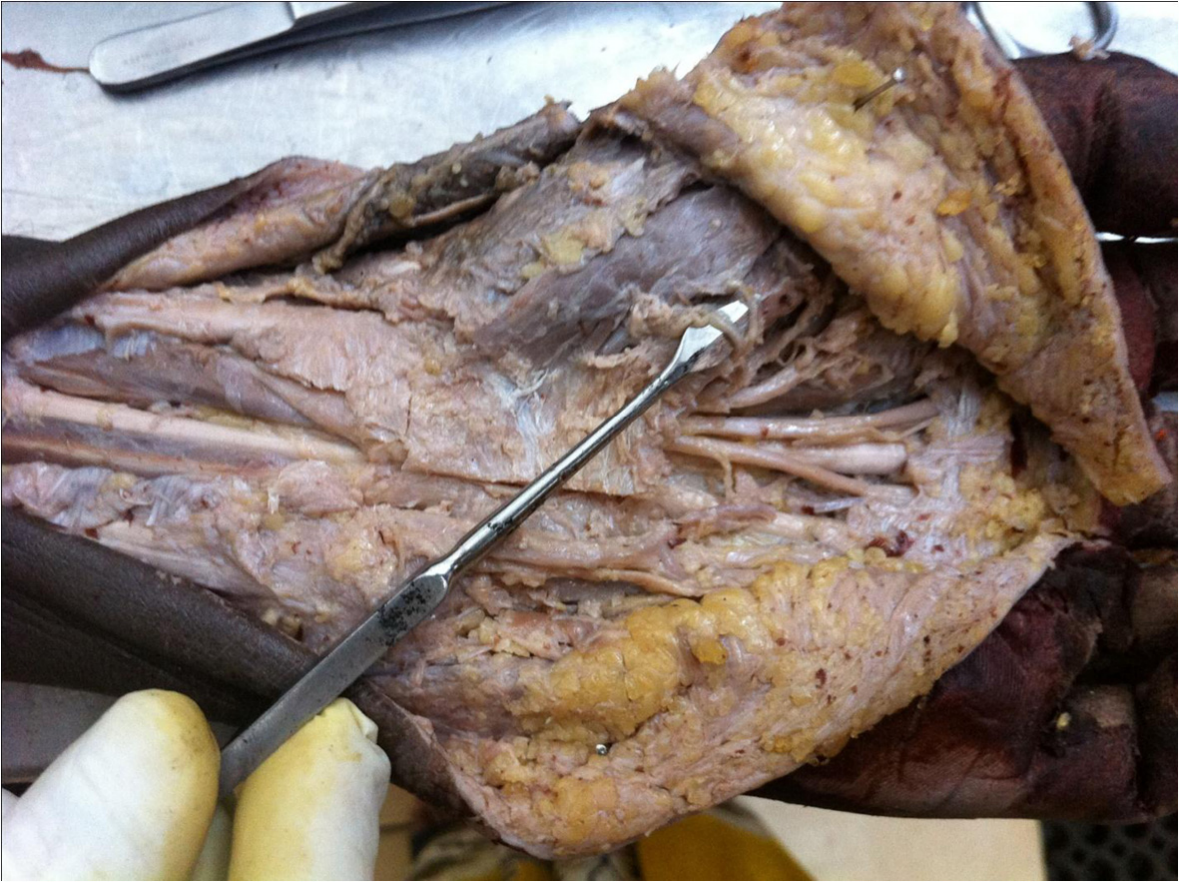
Dissected specimen showing multiple extra ligamentous recurrent branches of the median nerve (pointer).

**Figure 3:**
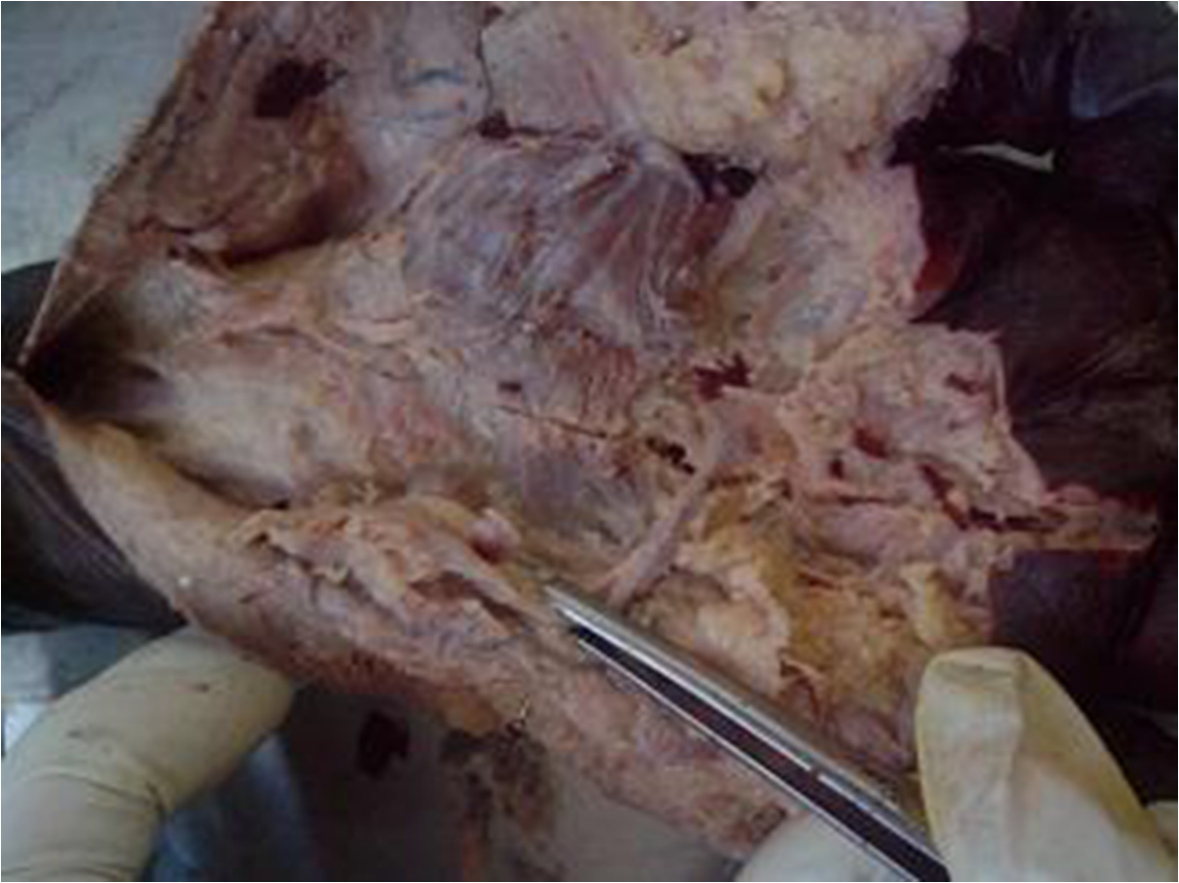
**Specimen with absent avascular plane along the TCL.** Thenar and hypothenar muscle attachments encroach upon each other across the midline.

**Figure 4:**
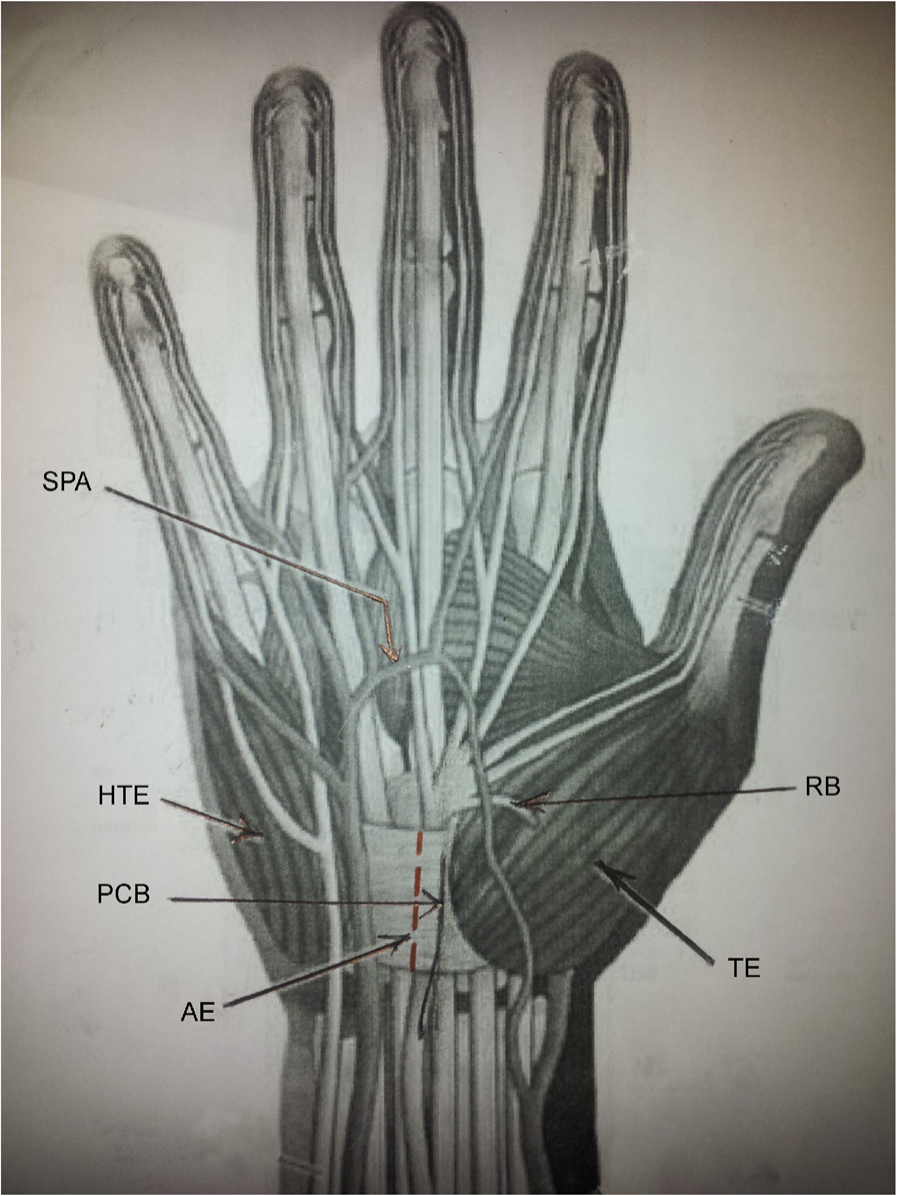
**Anatomical drawing to illustrate the key landmarks.** SPA - Superficial palmar arch, THE- hypothenar eminence muscle group, TE- Thenar eminence muscle group, RB- Recurrent branch of median nerve, PCB- Palmar cuatneous branch of median nerve, AE-Avascular area on the Transverse carpal ligament, free of muscle attachments. Proposed incision is marked in red, on the AE.

An incision was made on the palmar surface of each hand, along the plane of the radial border of the ring finger. The incision was then deepened to the plane of the transverse carpal ligament (TCL) and dissected carefully to expose the flexor retinaculum, the superficial palmar arch, the recurrent motor branch and the superficial palmar cutaneous branches of the median nerve (Figure 1, 2, 3). Following measurements were taken to the nearest 0.05 mm using a sensitive Vernier caliper (Figure 4).

● Distance to the palmar cutaneous branch of the median nerve from the incision at the proximal end of the transverse carpal ligament.

● Distance to the superficial palmar arch from the distal end of the transverse carpal ligament along the incision, in line with radial border of the ring finger.

● Length of the transverse carpal ligament along the radial border of the ring finger.

● Perpendicular distance from the above mentioned incision to the origin of the thenar motor branch of the median nerve. If there were several branches distance to the nearest branch was taken (Figure 3).

In addition to the above, we also attempted to describe an avascular area along which the transverse carpal ligament can be safely incised, in line with the initial incision along the radial border of the ring finger. We defined the avascular area as the portion of the transverse carpal ligament that is free of muscle attachments of the thenar and hypothenar eminences. The width of this area at the proximal distal ends as well as midpoint of the transverse carpal ligament were also measured.

The branching pattern of the thenar branch of the median nerve, whether it is single or multiple as well as whether it is extra ligamentous, trans ligamentous or sub ligamentous was also noted.

All data obtained were entered into a secure data base in the Statistical Package for Social Sciences (SPSS) version 20. Subsequently the data were analyzed using standard statistical software.

## Results (Table 1)

**Table 1:** Comparison of distance to the palmar cuatneous nerve and thenar branch from the relevant borders of PCL, and Length of the PCL measured along the incision

**Distance measured along the line of incision (mm)**	**Mean**	**Range**
To the palmar cutaneous nerve from proximal border of TCL	8.16	2.45–14.80
To the recurrent branch nerve from distal border of TCL	7.75	2.75–11.55
To the superficial palmar arch from distal border of TCL	11.48	4.00–18.25
Length of the TCL	27.00	20.60–34.25

Of the dissected 26 palms, majority (n = 16, 61.5%) were from male cadavers.

### Palmar cutaneous branch

Mean distance to palmar cutaneous branch from the proximal border of the transverse carpal ligament was 8.16 mm.

### Recurrnent thenar branch

In a majority of specimens the recurrent branch was a single trunk (n =20, 76.9%). Similarly 84.6% (n = 22) were extra ligamentous in location. Only 1 sub ligamentous specimen was noted (3.8%). 3 intra ligamentous specimens were also seen (1 1.5%)

Mean distance from the distal border of the transverse carpal ligament to the recurrent branch was 7.75 mm.

### Superficial palmar arch

Mean distance from the distal border of transverse carpal ligament to the superficial palmar arch was 11.48 mm.

### Length of the flexor retinaculum

Mean length of the flexor retinaculum, as measured along the incision, was 27.00 mm.

### Avascular area (Table 2 and 3)

**Table 2:** Mean width of the avascular area at the proximal, distal and midpoint of the avascular plane of the TCL, as measured along the initial incision of radial border of the index finger

**Point of measurement along the TCL**	**Mean distance mm**
Proximal	11.10
Mid point	8.04
Distal	7.09

**Table 3:** Mean width of the avascular plane at the proximal, distal and midpoint of the avascular plane of the TCL, as measured along the initial incision of radial border of the index finger

**Point of measurement along the TCL**	**Mean distance mm**	**Standard deviation mm (SD)**
Proximal	11.10	3.81
Mid point	8.04	3.33
Distal	7.09	3.62

Mean proximal and distal width of the avascular area was 11.10 mm and 7.09 mm respectively. In only one specimen (3.84%) the thenar and hypothenar muscle attachments merged into each other so that we could not identify the avascular area.

## Discussion

In a study conducted by Sacks it was noted that the average distance from the distal transverse carpal ligament to the superficial palmar arch was 18.8 +/− 0.6 mm and that to the thenar branch of the median nerve was 6.9 +/− 0.4 mm. The average length of the transverse carpal ligament was 28.5 +/− 0.8 mm [17].

Corresponding parameters in our study were noted to be 11.5 mm, 7.8 mm and 27.0 mm respectively (Table 1).

In our study majority of the thenar branches were extra ligamentous (84.6%). Similar results were obtained in other studies, where majority of nerves were classified as being extraligamentous [16-18]. In our study Sub ligamentous and intra ligamentous were 3.8% and 11.5% respectively. Corresponding parameters were 0% and 8% in one study [17] and 20% and 1.7% in another study [18]. Interestingly there is marked discrepancy in the sub and intra ligamentous variants, with reversed proportions in the above two studies. Our results are in more close agreement with Sacks et al. [17], where it was noted that the thenar branch of the median nerve was extra ligamentous in 92% and intra ligamentous in 8%. Nevertheless, there were no sub ligamentous branches noted in this study, which contrasts with our study, where nearly 4% were sub ligamentous.

The thenar branch of the median nerve contained one branch in 58% and multiple branches in 42% in one study [17]. Our study confirmed that a majority of the thenar branch was single, but the percentage was much higher in our series (76.9% Vs 58%).

In one study, the presence of hypertropic muscle on the transverse carpal ligament was noted to be associated with the trans ligamentous type, with the possibility of nerve branch passing within the muscle [16]. We did not come across such intramuscular thenar branches in our study.

In another study by Watchmaker et al., the course of the palmar cutaneous branch of the median nerve was studied in relation to the incision for carpal tunnel decompression. It was noted that incision placed approximately 5 mm ulnar to the interthenar depression, extending in the direction of the third web space, will decrease the incidence of injury to the palmar cutaneous branch [15]. Unfortunately the distance from the proximal border of the transverse carpal ligament to the palmar cutaneous branch was not measured in the above study.

In one study, the distance to the emergence of the palmar cutaneous branch was measured from the distal wrist crease, with mean distance being 2.09 +/− 0.31 cm [19]. In the above study, it was concluded that a longitudinal palmar incision could avoid injuries to the recurrent branch and palmar cutaneous branch of the median nerve and that an area of about 5 mm ulnar and 6 mm radial to the junction between the longitude of the third finger and distal skin crease at wrist level was a relatively safe area [19]. In another study, it was noted that an incision placed approximately 5 mm ulnar to the interthenar depression, pointing in the direction of the third web space, will decrease the incidence of injury to the palmar cutaneous branch of the median nerve [15]. Our study also revealed that the incision extending proximally more than 8.16 mm from the proximal border of transverse carpal ligament puts the palmar cutaneous branch at risk of being inadvertently divided.

Although bifid median nerve has been described as a cause for carpal tunnel syndrome [8], we did not come across this exceedingly rare variant in our series.

We attempted to describe an avascular area free of thenar and hypothenar muscle attachments on the transverse carpal ligament, along the proposed incision along the radial border of the ring finger. This was evident in all but one specimen (96.1%). Identification of such an avascular area will greatly aid safer decompression of the transverse carpal ligament along a bloodless field. We also noted that the proposed avascular area narrows distally as the mean width was lower distally than proximally. We propose that further large scale cadaveric and clinical studies should be carried out to further elaborate on this concept of an avascular area on the transverse carpal ligament.

## Conclusion

Careful attention to anatomical landmarks and meticulous surgical technique can minimize inadvertent damage to the palmar and thenar branches of the median nerve, as well as the superficial palmar arch, in carpal tunnel decompression.

We recommend an incision along the plane of the radial border of the ring finger for carpal tunnel decompression. Extending the incision more than 8.16 mm proximally and 7.75 mm distally from the corresponding borders of the transverse carpal ligament should be avoided at all costs, to prevent iatrogenic injury to the recurrent and palmar cutaneous branches of the median nerve respectively. Incision should be kept to a mean length of 27.0 mm, which corresponds to the length of transverse carpal ligament along the above axis.

Although rare, an intraligamentous or subligamentous thenar branch may suffer inadvertent damage when dividing the transverse carpal ligament. Although we could not find any constant association with hypertropic muscle over the transverse carpal ligament, with an intra- or sub ligamentous thenar branch, its presence should always be assumed and every precaution taken to preserve it.

Avascular area on the transverse carpal ligament, along the proposed incision along the radial border of the ring finger, should always be sought by the surgeon in carpal tunnel decompression. We suggest further large scale cadaveric and clinical studies to elaborate on this concept.

## Abbreviations

CTS: Carpal tunnel syndrome; CTD: Carpal tunnel decompression; TCL: Transverse carpal ligament.

## Competing interests

The authors declare that they have no competing interests.

## Authors’ contributions

MG, MJ and LS dissected the specimens and collected the data. LS analyzed the data, did the literature survey and prepared the manuscript. DA conceptualized and supervised the project. DA, APM and RJ contributed to critical revisions of the article. All authors read and approved the final manuscript.

## References

[B1] Atroshi I Gummesson C Johnsson R Ornstein E Ranstam J Rosen I Prevalence of carpal tunnel syndrome in a general population JAMA 1999 282 2 153 158 10.1001/jama.282.2.153 10411196

[B2] Jenkins PJ Srikantharajah D Duckworth AD Watts AC McEachan JE Carpal tunnel syndrome: the association with occupation at a population level J Hand Surg Eur Vol 2013 38 1 67 72 10.1177/1753193412455790 22832982

[B3] Atroshi I Gummesson C Johnsson R Sprinchorn A Symptoms, disability, and quality of life in patients with carpal tunnel syndrome J Hand Surg [Am] 1999 24 2 398 404 10.1016/S0363-5023(99)70014-6 10194028

[B4] Biazzo A Gonzalez Del Pino J Paralysis of the median nerve due to a lipofibrohamartoma in the carpal tunnel Rev Esp Cir Orthop Traumatol 2013 57 4 286 295 10.1016/j.recot.2013.04.005 23885655

[B5] Razik A Avisar E Sorene E Bilateral carpal tunnel syndrome with anomalous palmaris profundus tendons J Plast Surg Hand Surg 2012 46 6 452 453 10.3109/2000656X.2012.686914 23088639

[B6] Mayne AI Howard A Kent M Banks J Acute carpal tunnel syndrome in a patient with haemophilia BMJ case reports 2012 doi:10.1136/bcr-03-2012-6152 10.1136/bcr-03-2012-6152 PMC4543160 22761230

[B7] Ng CY Watts AC Acute carpal tunnel syndrome complicating a distal radial fracture in a patient with a persistent median artery J Hand Surg Eur Vol 2012 37 5 464 465 10.1177/1753193411436296 22311914

[B8] Granec D Bicanic G Boric I Delimar D Bifid median nerve in a patient with carpal tunnel syndrome–correlation of clinical, diagnostic and intraoperative findings: case report and review of the literature Acta Clin Croat 2012 51 4 667 671 23540177

[B9] Jenkins PJ Duckworth AD Watts AC McEachan JE Corticosteroid injection for carpal tunnel syndrome: a 5-year survivorship analysis Hand (N Y) 2012 7 2 151 156 10.1007/s11552-012-9390-8 23730233 PMC3351515

[B10] Ucar BY Demirtas A Bulut M Azboy I Ucar D Carpal tunnel decompression: two different mini-incision techniques Eur Rev Med Pharmacol Sci 2012 16 4 533 538 22696883

[B11] Acioly MA Maior PS Telles C Brasileiro de Aguiar G Bilateral Mini-Open Decompression in the Treatment of Carpal Tunnel Syndrome Caused by Persistent Median Artery: Case Report J Neuro Surg Part A, Cen Eur Neurosurg 2013 74 S 01 e124 e127 doi:110.1055/s-0032-1328959 10.1055/s-0032-1328959 23504667

[B12] MacDonald RI Lichtman DM Hanlon JJ Wilson JN Complications of surgical release for carpal tunnel syndrome J Hand Surg [Am] 1978 3 1 70 76 10.1016/S0363-5023(78)80118-X 621368

[B13] Kluge W Simpson RG Nicol AC Late complications after open carpal tunnel decompression J Hand Surg (Br) 1996 21 2 205 207 10.1016/S0266-7681(96)80098-2 8732401

[B14] Murphy RX Jr Jennings JF Wukich DK Major neurovascular complications of endoscopic carpal tunnel release J Hand Surg [Am] 1994 19 1 114 118 8169354 10.1016/0363-5023(94)90233-X

[B15] Watchmaker GP Weber D Mackinnon SE Avoidance of transection of the palmar cutaneous branch of the median nerve in carpal tunnel release J Hand Surg [Am] 1996 21 4 644 650 10.1016/S0363-5023(96)80019-0 8842959

[B16] Al-Qattan MM Variations in the course of the thenar motor branch of the median nerve and their relationship to the hypertrophic muscle overlying the transverse carpal ligament J Hand Surg [Am] 2010 35 11 1820 1824 10.1016/j.jhsa.2010.08.011 20934817

[B17] Sacks JM Kuo YR McLean K Wollstein R Lee WP Anatomical relationships among the median nerve thenar branch, superficial palmar arch, and transverse carpal ligament Plast Reconstr Surg 2007 120 3 713 718 10.1097/01.prs.0000270305.37677.e7 17700123

[B18] Mizia E Tomaszewski KA Goncerz G Kurzydlo W Walocha J Median nerve thenar motor branch anatomical variations Folia Morphol 2012 71 3 183 186 22936555

[B19] Xu X Lao J Zhao X How to prevent injury to the palmar cutaneous branch of median nerve and ulnar nerve in a palmar incision in carpal tunnel release, a cadaveric study Acta Neurochir 2013 155 9 1751 1755 10.1007/s00701-013-1764-3 23828713

